# The effect of doorway characteristics on freezing of gait in Parkinson’s disease

**DOI:** 10.3389/fneur.2023.1265409

**Published:** 2023-12-04

**Authors:** Helena M. Cockx, Eefke M. Lemmen, Richard J. A. van Wezel, Ian G. M. Cameron

**Affiliations:** ^1^Department of Neurobiology, Faculty of Science, Donders Institute for Brain, Cognition and Behaviour, Radboud University, Nijmegen, Netherlands; ^2^Department of Neurology, Center of Expertise for Parkinson and Movement Disorders, Donders Institute for Brain, Cognition and Behaviour, Radboud University Medical Center, Nijmegen, Netherlands; ^3^Biomedical Signals and Systems Group, Faculty of Electrical Engineering, Mathematics and Computer Science, University of Twente, Enschede, Netherlands; ^4^OnePlanet Research Center, Nijmegen, Netherlands; ^5^Faculty of Science, Education Center, Radboud University, Nijmegen, Netherlands

**Keywords:** Parkinson’s disease, gait, surveys and questionnaires, freezing of gait, doorways

## Abstract

**Background:**

Freezing of gait is a debilitating symptom in Parkinson’s disease, during which a sudden motor block prevents someone from moving forward. Remarkably, doorways can provoke freezing. Most research has focused on the influence of doorway width, and little is known about other doorway characteristics influencing doorway freezing.

**Objective:**

Firstly, to provide guidelines on how to design doorways for people with freezing. Secondly, to compare people with doorway freezing to people without doorway freezing, and to explore the underlying mechanisms of doorway freezing.

**Methods:**

We designed a web-based, structured survey consisting of two parts. Part I (*n* = 171 responders), open to people with Parkinson’s disease with freezing in general, aimed to compare people with doorway freezing to people without doorway freezing. We explored underlying processes related to doorway freezing with the Gait-Specific Attention Profile (G-SAP), inquiring about conscious movement processes occurring during doorway passing. Part II (*n* = 60), open for people experiencing weekly doorway freezing episodes, inquired about the influence of specific doorway characteristics on freezing.

**Results:**

People with doorway freezing (69% of Part I) had higher freezing severity, longer disease duration, and scored higher on all sub scores of the G-SAP (indicating heightened motor, attentional, and emotional thoughts when passing through doorways) than people without doorway freezing. The main categories provoking doorway freezing were: dimensions of the door and surroundings, clutter around the door, lighting conditions, and automatic doors.

**Conclusion:**

We provide recommendations on how to maximally avoid freezing in a practical setting. Furthermore, we suggest that doorways trigger freezing based on visuomotor, attentional, and emotional processes.

## 1 Introduction

Every day, we pass doorways in various dimensions and with various appearances, without realizing that crossing these doors can be a real struggle for people with Parkinson’s disease. People with Parkinson’s disease may experience sudden motor blocks, a phenomenon which is called freezing of gait, when attempting to pass through a doorway (1). Besides at doorways, freezing can also occur during turning, gait initiation, dual-tasking (e.g., talking while walking), or while being under time pressure (2, 3). These freezing episodes are very debilitating and can lead to falls and hospital admissions (4, 5).

Previous research investigating the phenomenon of doorway freezing has mainly focused on the width of doorways (6–12). These studies showed that people with freezing adjusted their gait pattern more and experienced more freezing when passing narrow doorways than when passing wider doorways (6–8, 11). These authors attributed this finding to an inability to appropriately scale their movements toward the upcoming doorway obstacle due to a mismatch between visual and proprioceptive information causing problems with motor planning (6, 9, 12–14). Alternatively, a narrow doorway may be perceived as distracting or threatening, drawing attention away from the walking task, hence deteriorating gait and triggering freezing (15, 16).

Although these previous laboratory studies indicate that doorway width seems to be a substantial contributor to triggering freezing, other factors may play a role too. When we designed a narrow doorframe to trigger freezing for a previous laboratory study (17), this doorway failed to induce freezing in multiple participants, even though they displayed doorway freezing at other doors leading to the experimental set-up. Anecdotal reports from these patients informed us that other characteristics of doorways might influence their freezing severity, such as a change in flooring from one room to another, or the change in lighting between two rooms.

While other trigger types (e.g., turning or dual-tasking) have been extensively investigated (2, 18), the characteristics of doorways that trigger freezing largely remain an unexplored area. Identification of contributing factors that trigger doorway freezing may help to design places like care homes, hospitals, and people’s homes in a way where the chances of freezing are as small as possible. Conversely, since triggering freezing in a lab setting is a difficult undertaking given participants’ heightened levels of attention and/or stress (19), identification of these contributing factors may help researchers optimally design future lab settings to maximally trigger freezing. Furthermore, understanding what exactly triggers people to freeze when walking through doorways can also help further our understanding of the remarkable phenomenon.

Our primary goal was to provide guidelines on how to optimize the home environment for people with freezing (and how to set up studies to investigate doorway freezing). Therefore, we designed a survey inquiring about different doorway characteristics and whether they aggravate or ameliorate freezing. We hypothesize that doorway width will be a factor that influences doorway freezing, but that other doorway characteristics will play a – potentially larger – role. We had two secondary aims. Firstly, to compare disease and population characteristics between people with Parkinson’s disease *with* freezing at doorways (“doorway freezers”) to people with Parkinson’s disease *without* freezing at doorways. Secondly, to explore whether doorway freezing indeed relates to dysfunctional visuomotor processing as mainly hypothesized in literature, or whether disturbed attentional processes due to cognitive or affective distractors also play a role. Therefore, we added a questionnaire inquiring about various types of mental processes occurring during doorway passing (the Gait-Specific Attention Profile, G-SAP) (20, 21).

## 2 Materials and methods

### 2.1 Survey

We set up a web-based, structured survey consisting of two parts. Part I was open to participants aged 18 and older with a self-reported diagnosis of Parkinson’s disease and self-reported freezing. It was intended to address the two secondary research goals, namely, to characterize the population of doorway freezers compared to non-doorway freezers, and to explore the underlying mechanisms of doorway freezing by asking participants about their conscious movement processes when walking through doorways with the G-SAP. Part II was only open for doorway freezers (minimally once per week) and addressed the primary research goal, namely, to identify doorway characteristics that aggravate or ameliorate freezing.

For the first part (± 10 min), participants answered disease-related (e.g., years since diagnosis) and demographic questions (e.g., age, sex). The remainder of Part I was made up of: the Dutch version of the new freezing of gait questionnaire (NFOGQ) (22), which was supplemented with two questions in the same style and wording about freezing when walking through a doorway (e.g., “How often do you experience freezing episodes when walking through a doorway?”); and an adapted, Dutch translation of the G-SAP (20), during which we inquired about conscious movement processes when walking through a doorway. The G-SAP is a questionnaire designed to assess conscious mental processes during gait. We adapted this questionnaire to inquire about mental processes specifically when walking through doorways. The G-SAP consists of 11 questions, which contribute to four sub scores: conscious movement processing (three questions), processing inefficiencies (two questions), anxiety (three questions), and fall-related ruminations (three questions). Each question scores between 1 and 5 points depending on how strongly participants agree with statements about how they feel when walking. We hypothesized that if doorway freezing is indeed related to visuomotor dysfunction, doorway freezers would score higher on the conscious movement processing G-SAP sub score when walking through doors (e.g., “I think about the way I walk”) compared to non-doorway freezers, indicating that they consciously monitor their movements to compensate for impaired visuomotor capabilities. Furthermore, if freezers score higher on the processing inefficiency (e.g., “I find it difficult to concentrate on two things at once”) G-SAP sub score, this would indicate an attentional deficit related to doorway freezing. Similarly, higher scores on the anxiety (e.g., “I feel strained when walking through doors”) or on the fall-related ruminations (e.g., “I think about falling when walking through doors”) G-SAP sub score, would argue for an emotional mechanism underlying doorway freezing.

**Figure 1:**
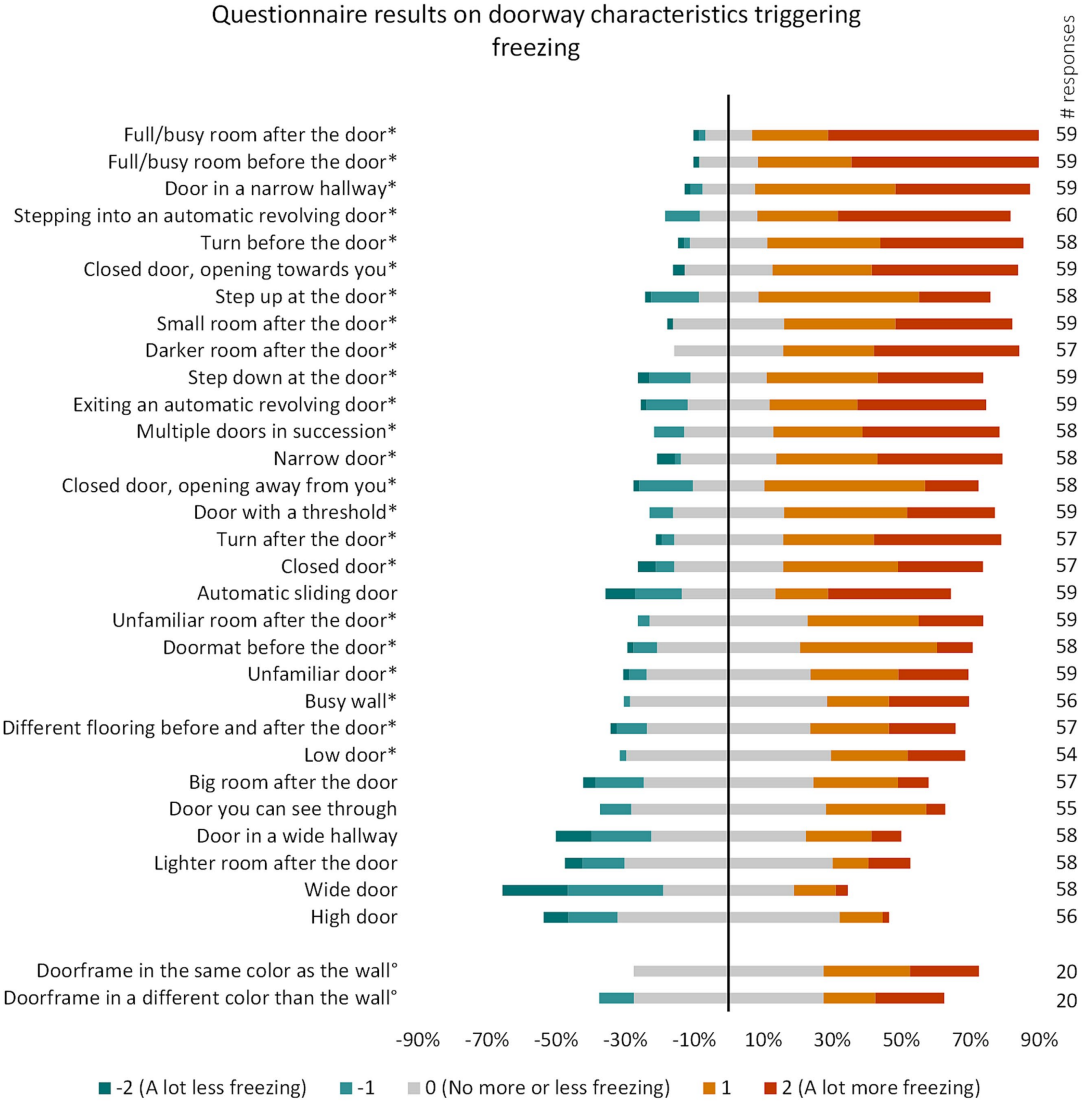
Part II questionnaire results per doorway characteristic sorted from highest to lowest number of respondents indicating an increase in freezing. These questions were not mandatory, number of respondents per question are indicated in the column on the right. *p < 0.001 (*p* < 0.05 Bonferroni-corrected for 32 comparisons). °These questions were added in a later stage and therefore have fewer responses.

If participants indicated that they had freezing at doorways at least once per week, they were invited to participate in the second part of our survey, which was sent to them 1–2 weeks after completing Part I. In the meantime, we asked them to pay attention to which doorways triggered their freezing more than normal, and which doorways caused less freezing than normal. Normal was defined to them as an average, open door. We encouraged them to take photographs of these doors and to talk to people in their environment to help them prepare for the questionnaire, but these photographs were not uploaded as part of the survey nor shown to the researchers for privacy reasons. Part II of the questionnaire (± 20 min) contained 30 questions about doorway characteristics that could potentially influence freezing behavior, e.g., wide and narrow doorways, dark or light room after the door, etc. (see Supplementary material or Figure 1 for the full list). This list of characteristics was based on anecdotal reports from participants in one of our previous studies about freezing at doorways (17), as well as information from the participants of a focus group. We asked all of these people what caused them to freeze at doorways, and created a list of doorway characteristics based on their answers. For each characteristic, we asked participants to indicate whether they experienced less, the same amount, or more freezing on a scale of −2 to +2, with −2 indicating much less freezing, 0 indicating the same amount of freezing, and + 2 indicating much more freezing compared to an average, open door. At the end of the survey participants had the opportunity to share – in an open-ended question – other doorway characteristics influencing their freezing severity that were not yet mentioned. Participants could leave questions open if they did not know what influence a certain doorway characteristic had. They were encouraged to consult people in their environment to help them with answering the questions. Both parts of the questionnaire are available in Supplementary material.

The survey was developed in collaboration with a focus group of three people with Parkinson’s disease who experience doorway freezing daily, and two patient-researchers with Parkinson’s disease. The project received ethical approval from the Research Ethics Committee of the Faculty of Science of the Radboud University (REC22102). All participants provided informed consent before filling in the survey.

The questionnaire was sent out to 2,538 people of the database of ParkinsonNEXT with Parkinson’s disease (freezing-status unknown) and was openly accessible through the ParkinsonNEXT website. ParkinsonNEXT^1^ is a Dutch online platform that aims to connect researchers to people with Parkinson’s disease and their caregivers in The Netherlands. The questionnaires were accessible between August 31^st^, 2022, and December 21^st^, 2022. Participants aged 18 and older with a self-reported diagnosis of Parkinson’s disease and self-reported freezing could participate in the questionnaire.

### 2.2 Statistics

The following data was excluded from analysis: responses from people with parkinsonism (*n* = 10), incomplete responses from Part I (*n* = 8), incomplete responses from Part II, except when missing only one value (*n* = 3), incomplete responses on the G-SAP score (*n* = 3).

All statistical analyses were performed with IBM SPSS 28 with a significance level set at 0.05. For Part I, we compared demographical and clinical characteristics of people that reported doorway freezing at least once per month with people that did not report doorway freezing with two-sample *t*-tests for continuous variables and *χ*^2^ for discrete variables. We performed the same tests to assess differences between responders and non-responders of Part II. G-SAP scores were calculated as a percentage of the maximum score for each category, with 0% representing a G-SAP score of 0, and 100% representing the maximum score. We compared G-SAP sub scores between groups with different frequencies of doorway freezing (no freezing, once per month, once per week, once per day, and more than once per day) with a Kruskal-Wallis test, which was corrected for multiple comparisons for all four sub scores. *Post-hoc*, we compared the group with no freezing at doorways to each group with freezing at doorways of a different frequency with a Dunn test with Bonferroni correction for multiple comparisons for each of the four comparisons.

For the results of Part II of the questionnaire, we performed a one-sample Wilcoxon signed rank test on each doorway characteristic to verify whether the median score differed significantly from 0. Bonferroni correction was done for multiple comparisons (*n* = 32).

## 3 Results

### 3.1 Study population

**Figure 2:**
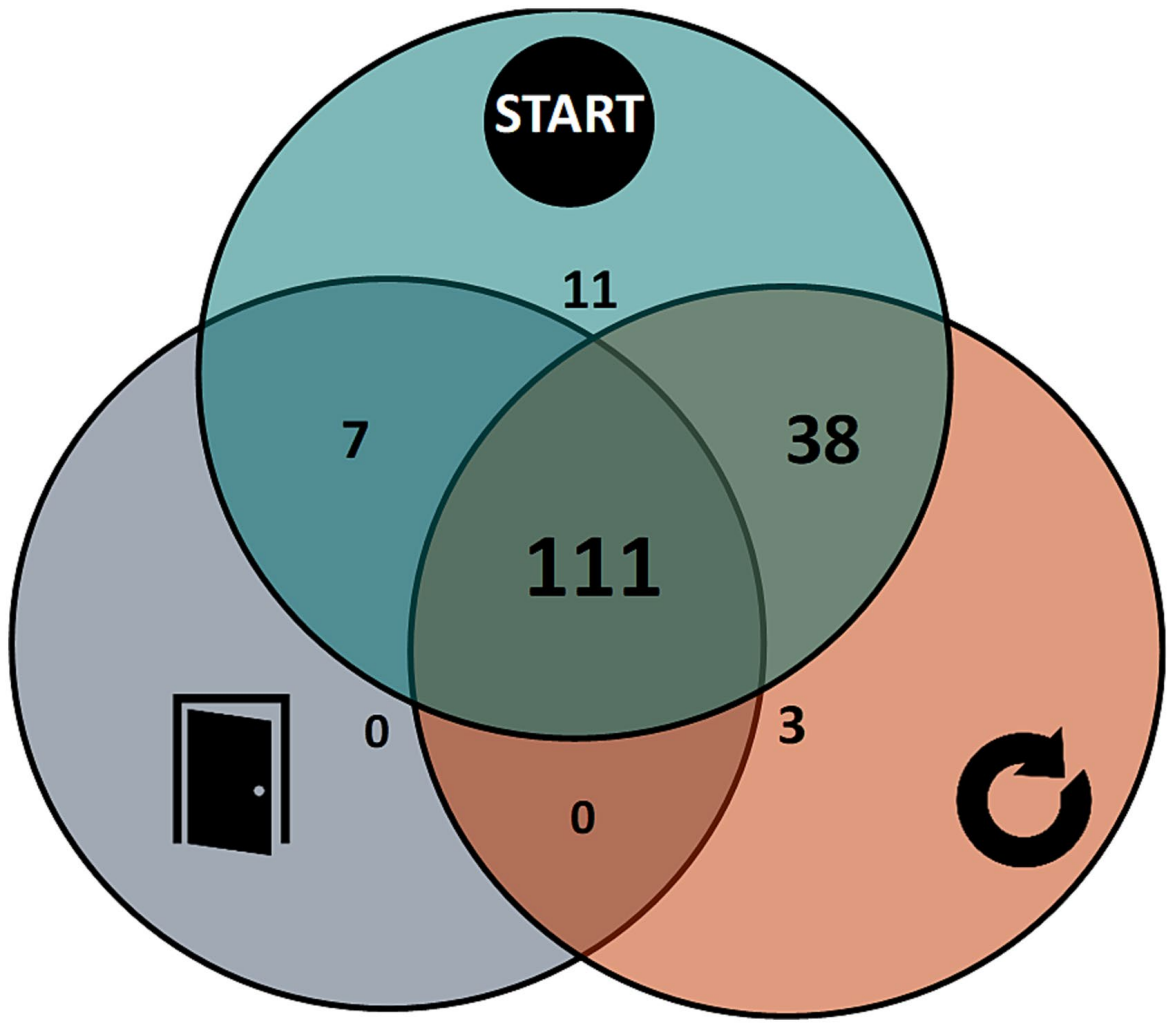
Distribution of respondents that indicate experiencing each possible combination of freezing triggers (start, turn, and doorway freezing). One respondent indicated having freezing, but not for any of these specific triggers.

Table 1 displays the demographical and clinical characteristics of the respondents for both parts of the questionnaire. A total of 171 people filled in Part I of the questionnaire of which 118 (69.0%) indicated experiencing freezing at doorways at least once per month. Statistical comparisons between the participants with and without freezing at doorways revealed that participants with freezing at doorways had a significantly higher NFOGQ score (*p* = 0.002) and a longer time since diagnosis (*p* = 0.002). No statistically significant differences in age, gender, or freezing frequency were found between the two groups. Figure 2 shows the proportion of the respondents that reported freezing during gait initiation, turning, doorway passing, or any combination of these triggers. The vast majority of the participants (64.9%) reported freezing for all three triggers. The most common trigger for freezing was gait initiation (97.7%), followed by turning (88.9%) and doorways (69.0%). Remarkably, 94.1% (111/118) of doorway freezers had freezing for all three triggers, whereas only 66.5% (111/167) of gait initiation freezers and 73.0% (111/152) of turning freezers had freezing for all triggers. Doorway freezing was the only type of freezing not seen in isolation.

**Table 1:** Population characteristics.

	Part I				Part II
	No freezing at doorways	Freezing at doorways	value of *p*	Total	Total
*N* (%)	53 (31%)	118 (69%)		171	60
Age (years)	67.6 ± 8.8	69.2 ± 8.5	0.265	68.7 ± 8.6	68.0 ± 9.4
Time since diagnosis (years)	8.7 ± 5.0	11.4 ± 5.3	0.002*	10.5 ± 5.3	11.6 ± 5.1
Sex (% man)	60.4%	72.0%	0.129	68.4%	76.7%
NFOGQ (median [range])	15 [4–23]	19 [8–25]	0.002*	18 [4–25]	18 [9–25]

Values are represented as mean ± sd, unless otherwise specified. Only participants who had freezing at doorways at least once per week were eligible for part II.

*p < 0.05 between respondents with and without freezing at doorways as determined by *t* test (means) or *χ*^2^ test (proportions and medians).

Out of the 97 participants that were eligible for the second part of the questionnaire (experiencing doorway freezing at least once per week and having consented to the follow-up survey), 60 filled out Part II. The 37 non-responders were not significantly different from the responders regarding sex, time since diagnosis, freezing frequency, and NFOGQ score, but were significantly older compared to responders (mean ± SD: 72.17 ± 6.6 vs. 68.0 ± 9.4 years old; *p* = 0.025).

### 3.2 Gait-specific attention profile (G-SAP)

**Figure 3:**
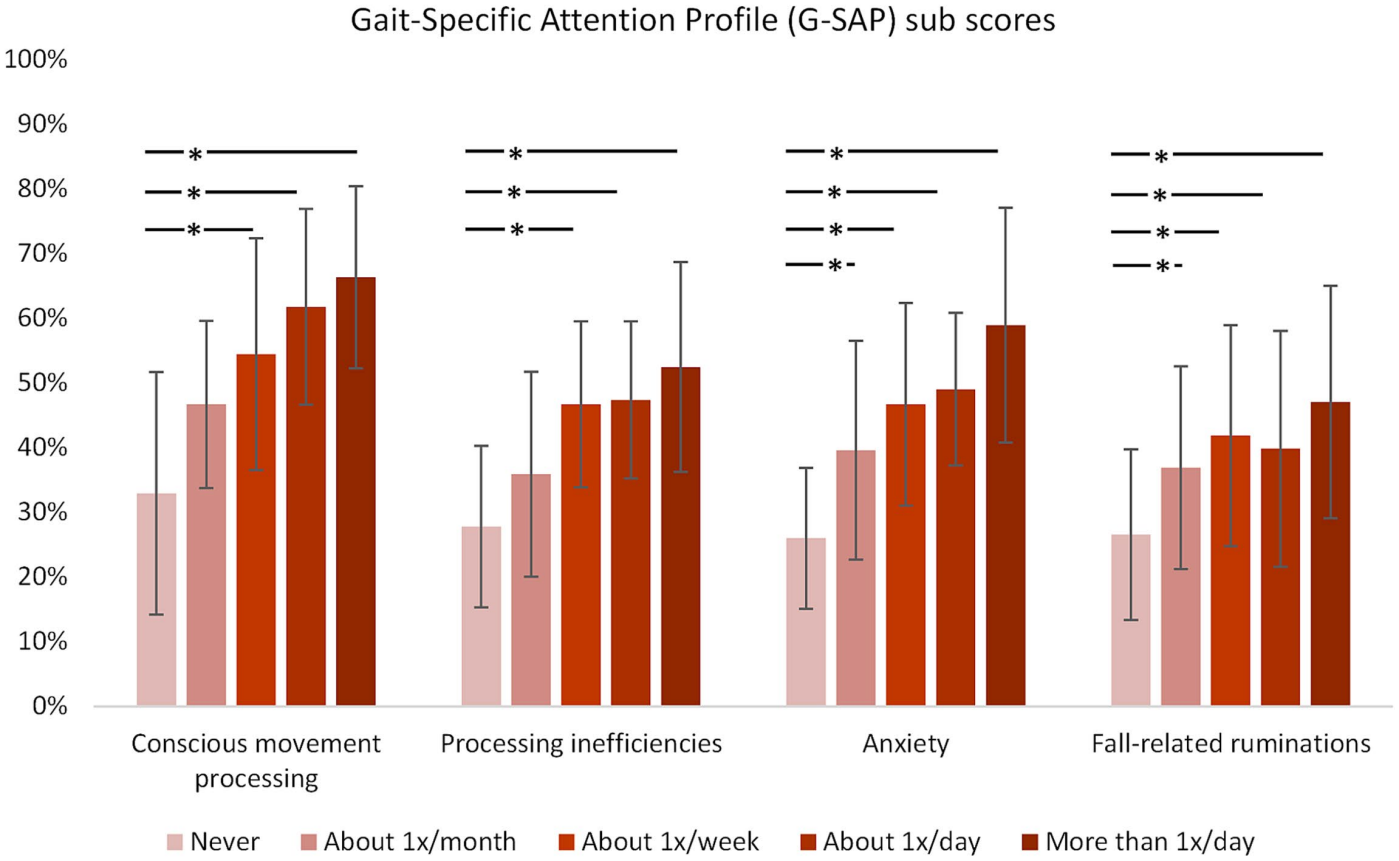
Results for the G-SAP sub scores for respondents with different frequencies of doorway freezing. *p < 0.0125 (*p* < 0.05 Bonferroni-corrected for 4 comparisons). Error bars indicate standard deviation.

Figure 3 shows all sub scores for the adapted version of the G-SAP for doorway passage, which assessed various mental processes when walking through a door. As a reminder, these various sub scores of the G-SAP relate to either conscious movement processes (e.g., thinking about the way you walk), processing inefficiencies (e.g., having difficulties focusing on two things at once), anxiety (e.g., feeling strained), or fall-related ruminations (e.g., thinking about what would happen if you fell).

The Kruskal-Wallis test indicated significant differences between the groups for all sub scores (all *p* < 0.001). *Post hoc* analysis between the group without freezing at doorways and each group with freezing at doorways at different frequencies revealed significant differences for all sub scores between the group without doorway freezing and the groups that reported doorway freezing *at least* once per week. An additional statistically significant difference was found between the group without doorway freezing and the group that reported doorway freezing once per month for anxiety and fall-related ruminations sub scores.

### 3.3 Doorway characteristics influencing freezing

Figure 1 contains an overview of all doorway characteristics from Part II of the questionnaire. We displayed the doorway characteristics that increased freezing frequency in most of our participants at the top. Out of the 32 doorway characteristics from the survey, 23 had a significant negative influence on freezing frequency (*p* < 0.001 due to correction for multiple comparisons (*n* = 32)).

A full or busy room after or before the door were the factors that aggravated doorway freezing in most of the respondents (83.1 and 81.4%, respectively). This was followed by: a door in a narrow hallway (79.7%) and stepping into an automatic revolving door (73.3%). In contrast, the doorway characteristics that *decreased* freezing severity in most of the respondents were: wide doors (46.6%), doors in a wide hallway (27.6%), and automatic sliding doors (22.0%).

No formal analysis was performed on the open questions. In total, 33 participants input text after the question: “Are there any other doorway characteristics that influence your freezing?” From these answers, we found 19 specific doorway characteristics that were mentioned by participants. The two most common answers (both *n* = 4) were a resistive door closing system and people or objects near the door, which is something we already inquired about in the survey in various ways. An overview of all doorway characteristics mentioned by participants can be found in Supplementary Table 1.

Taken together, we identified the following categories that influenced freezing behavior: dimensions of the door and the environment, clutter around the door, lighting conditions, and automatic doors.

## 4 Discussion

We conducted a web-based, structured survey to systematically investigate characteristics of doorways that aggravate or ameliorate freezing behavior. In total 69.0% of the 171 respondents (all self-reported freezers) reported experiencing doorway-provoked freezing, which is in line with previous reports (23). Doorway freezers had higher freezing severity, longer disease duration, and scored higher on all sub scores of the G-SAP than people without doorway freezing. Sixty respondents who reported to have doorway-provoked freezing at least once per week filled in the second part of the questionnaire. As hypothesized, the width of the doorway was not the only - and not the most important - factor influencing freezing behavior. Below, we zoom in on a few doorway characteristics that were brought forward by our survey and provide practical tips on how to avoid freezing in daily life. The same tips can be applied in reverse to maximally trigger doorway freezing in an experimental setting. Next, we explore what the results of this survey can teach us about the underlying mechanisms of doorway freezing.

### 4.1 Factors that influence freezing behavior

#### 4.1.1 Dimensions of the door and the environment

Consistent with previous literature, narrow doorways, narrow hallways, and small rooms after the door increased the likelihood of freezing (6–8, 10, 11). Note that narrow hallways (10) (third most influential factor) negatively affected freezing severity in even more participants than narrow doors (ranked 13th). In contrast, wide doorways scored the highest number of participants indicating a decrease in freezing severity out of all doorway characteristics. Not only the width of the door played a role, but also the height, with lower doorways significantly increasing the risk of freezing.

The influence of doorway dimensions on freezing is in line with the theory that doorway freezing results from inappropriate scaling of motor responses to visuo-proprioceptive inputs, possibly due to a deficit in motor planning (6, 9, 12–14). However, narrow hallways and small rooms after the door may also act as an anxiety-inducing trigger as they may feel oppressive (24).

**Recommendation**: In practice, when designing care homes and hospitals for people with freezing, we recommend to avoid small and low doorways as much as possible and provide wide and open spaces before and after doors.

#### 4.1.2 Clutter

Remarkably, the amount of clutter before and after the door were the highest-ranking factors influencing freezing behavior, increasing the chances of freezing in 81.4 and 83.1% of the respondents, respectively. This observation is in line with a previous survey study, reporting that a cluttered environment was the second most important triggering factor when inquiring about triggers for freezing in general, only being exceeded by gait initiation, which was also the most common triggering factor for Part I of our survey (25). Another survey-based study reported a lower percentage (46.4%) of cluttered places triggering freezing (26). This discrepancy may relate to our cohort of doorway freezers having a higher freezing severity than the cohort from the previous study (being general freezers), or that clutter and doorways may induce freezing through similar mechanisms.

The high influence of clutter on doorway freezing suggests that attentional processes play an additional role as a causal mechanism for doorway freezing. A previous study investigated visual search behavior in freezers when walking toward a doorway in a cluttered environment (21). They found that freezing episodes were related to a prolonged eye fixation toward the obstacles while focusing on the walkway seemed to prevent freezing from manifesting. Similarly, another study reported that when not freezing, freezers fixated their gaze on the walking path instead of on the doorway, probably reflecting a freezing-preventive strategy (16). Although clutter seems to be an important factor in freezing behavior, relatively few studies have investigated this type of trigger and future studies should further explore the mechanisms behind this phenomenon.

**Recommendation**: From a practical point of view, removing clutter around doorways and keeping the space around doors as open and tidy as possible is a relatively easy-to-apply measure to prevent freezing.

#### 4.1.3 Lighting conditions

Another influencing factor, and one that is relatively easy to adjust in a practical setting, is the difference in lighting before and after the door. A darker room after the door increased freezing frequency in the majority of the respondents (68.4%) and it is the only doorway characteristic for which none of our respondents reported a decrease in freezing severity. A lighter room after the door, in contrast, was fifth on the list of factors that decreased freezing frequency in most participants our survey. This result is consistent with a previous study observing more freezing when people walked toward a doorway in complete darkness than in daylight (15). Freezing severity in this study further decreased when the door frame and the limbs were illuminated. It is important to consider that according to our results the room after the door does not have to be completely dark, but being relatively darker may already lead to an aggravation of freezing.

This observation suggests that anxiety-related factors may also be involved in doorway freezing, as darker rooms can feel more threatening than lighter rooms (24). However, a sensory-processing component of darker rooms causing freezing cannot be excluded (15).

**Recommendation**: Ensuring that rooms are always well-lit *before* entering a room can be of assistance. This can easily be implemented by adding a light switch in the hall or increasing the sensitivity of motion sensors that turn on the lights.

#### 4.1.4 Automatic doors

In general, all types of automatic doors increased freezing severity in more than half of our participants with ‘stepping into an automatic revolving door’ (73.3%) and ‘exiting an automatic revolving door’ (62.7%) scoring highest in our survey, followed by ‘walking through an automatic sliding door’ (50.8%). Note that automatic sliding doors also improved freezing in 22.0% of the respondents, probably because they remove the necessity of an additional action to open the door. Therefore, the influence of automatic sliding doors on doorway freezing was not statistically significant, whereas both entering and exiting automatic revolving doors caused a significant increase in freezing frequency. Our results are consistent with previous studies reporting that automatic sliding doors (such as the ones from an elevator) induce freezing in 40–50% of the freezing respondents (23, 26).

Automatic doors probably aggravate freezing severity by adding time pressure to the gait task, and – in case of revolving doors – by imposing a specific gait speed and walking path.

**Recommendation**: When designing care homes, clinics, or other public buildings buildings where the use of automatic doors cannot be avoided, we recommend opting for automatic sliding doors that stay open as long as someone is close to the door, instead of for automatic revolving doors.

#### 4.1.5 Extra factors

A few other interesting doorway characteristics that can help to avoid freezing were brought up by the open-ended questions at the end of our survey. Several respondents (*n* = 4) mentioned that closing systems that create resistance or heavy doors are an aggravating factor for freezing, requiring extra force to push and hold the door open. Adding an automatic opening-and-closing mechanism (that holds the door open as long as someone’s near) or choosing lighter doors, can be a good solution. The color of the doorframe and the wall around it may be an additional influencing factor and we added this question to the questionnaire at a later stage. Indeed, if the doorframe has the same color as the wall, this increased freezing for more of our respondents than if the doorframe has a different color. This finding suggests that more salient doors may decrease freezing severity, which is a relatively easy measure to apply with a lick of paint.

#### 4.1.6 Practical implications

In Table 2, we summarize a few tips which can be applied when designing homes, hospitals, care homes, or public spaces to minimize the likelihood of freezing. When designing a laboratory setup intended to provoke freezing as much as possible for research purposes, we advise to create a setup by using the reverse of the tips (e.g., use narrow and low doors). Of note, the factors influencing freezing behavior can be very personal and we advise discussing possible adjustments with the end-users to find tailor-made solutions, especially when it comes to applying changes in the at-home situation. Based on our experience from this survey, we conclude that people are in general very aware of the factors that influence their freezing behavior.

**Table 2:** Recommendations regarding freezing of gait at doorways.

How to minimize freezing at doorways?
High and wide doorways and hallways are preferable Make sure rooms are well lit before entering (e.g., a light switch or sensor before entering) Avoid clutter around doors When necessary, automatic sliding doors are preferable over automatic revolving doors Avoid resistive door closing systems (e.g., stiff springs, hydraulic systems)

Remarkably, some doorway characteristics revealed a group of respondents reporting less freezing related to a certain factor, while another group reported more freezing. This was in contrast to other characteristics that showed a clearly skewed distribution toward either a positive or negative response. For instance, some people benefitted from a step at the doorway, probably working as a kind of cue (27, 28), while others’ freezing got worse with a doorway step, potentially induced by a fear of tripping (4, 5). In another example, some participants indicated that wide doorways decreased their freezing as expected, while others reported an increase in freezing, which could be due to the fact that a wider doorway is harder to use as a support while walking through it. In a similar vein, most respondents reported more freezing when opening a door away from them, probably due to the extra action, but some reported less freezing, possibly because the door can serve as extra support. Both the doorpost and door handle acting as support and the threshold acting as a cue were mentioned by respondents in the open questions. As mentioned before, a similar effect is seen with automatic sliding doors, which can aggravate freezing because of the added time pressure, but also alleviate freezing because they automatically open up the walkway taking away the additional action involved in opening a door by hand.

### 4.2 Exploring the mechanisms behind doorway freezing

Typically, doorways are thought to trigger freezing due to impaired visuomotor processes (6, 9, 13, 14). However, some researchers argue that doorway freezing may also be caused by attentional or emotional processes leading to distraction from the gait task (15, 16). This study reported various doorway characteristics that can be attributed to visuomotor, as well as attentional or emotional processes: narrow and low doors could act as visuomotor triggers, cluttered spaces as attentional distractors, and darker and smaller rooms as anxiety-provoking factors. However, it is difficult to precisely determine why a certain doorway characteristic increases the chance of freezing purely based on this survey, and future research will be necessary in order to shed more light on this.

The analysis of the G-SAP scores similarly indicates that doorway freezing seems to not only relate to dysfunctional visuomotor processes but also to attentional and emotional processes. People with doorway freezing scored higher on all sub scores of the G-SAP (conscious movement processing, processing inefficiencies, anxiety, and fall-related ruminations) and these sub scores increased systematically with increasing frequency of doorway-provoked freezing, hence reflecting more conscious thought processes with regards to visuomotor, attentional, and emotional aspects of doorway passing.

In general, we observed that doorway freezers had significantly worse freezing severity (based on the NFOGQ) and longer disease duration than people without doorway freezing. Furthermore, all people that reported doorway freezing also reported having starting hesitation (*n* = 118) and almost all having turning freezing (*n* = 111). No one reported having doorway freezing in isolation. This observation suggests that doorway freezing is a more severe type of freezing, rather than a separate entity with a different causal mechanism. However, future research should further investigate how doorway freezing exactly relates to the other types of freezing.

Taken together, our results suggest that doorways do not solely trigger freezing based on visuomotor processes, but that attentional and affective processes are likely to be involved as well. These underlying mechanisms to doorway freezing can differ depending on the circumstances, and may not be the same across all freezers. The fact that some factors were reported to increase freezing in some people but decrease it in others illustrates person-specific effects. Although we provide suggestions about the underlying processes related to doorway freezing, the results of this survey should be interpreted with caution, and future experimental studies are required to further investigate this topic.

### 4.3 Study limitations

Survey studies are never without limitations and our results should also be interpreted with care. Firstly, sampling bias and non-response may result in biased outcomes. We verified that Part I of our survey was filled in by a representative sample (*n* = 171) of the ParkinsonNEXT cohort (*n* = 2,538). Our study respondents were of similar age and sex as the cohort, however, they had longer disease durations, probably reflecting that freezing usually manifests at a later stage in the disease (29). Because our survey was split into two parts to give people time to prepare for filling out Part II, we were able to characterize the participants who were eligible for Part II, but failed to respond. Analysis of the responders and non-responders revealed that there were no significant demographic or clinical differences between these two groups, except that the non-responders were on average slightly older. This may reflect that the older people encountered more difficulties in filling in the web-based survey.

Secondly, recall bias is another common bias in survey studies, and self-reported freezing has been considered to not reliably reflect actual freezing severity (22, 30). We have tried to address this issue by sending Part II of the survey 1–2 weeks after the completion of Part I. In the meantime, we asked participants to reflect on doors that influence their freezing, to take pictures of these doors, and to discuss their difficulties with relatives and friends. Furthermore, we encouraged participants to leave open any questions on doorway characteristics if they were not sure about the effect of that characteristic in order to prevent participants from ‘guessing’ answers. We did not include photographs in the survey, because although real-life pictures could possibly help with visualization, it is impossible to provide photographs where only the indicated characteristic changes. For instance, the lighting condition or color scheme of a door may also be different in different pictures.

A last potential limitation of this study was the scoring system that was used to inquire about the influence of various doorway characteristics on freezing severity. We asked participants to score each doorway characteristic on a scale from −2 to +2, indicating whether the characteristic induced less or more freezing than a standard door. This might not be a very intuitive way to score freezing. However, before sending out the survey, we evaluated the scoring system with a patient focus group and two patient-researchers who determined the questions as understandable. Furthermore, the observation that narrow doors induced more freezing and wider doors induced less freezing was in line with previous literature, suggesting that the questions and answering possibilities were interpreted correctly.

### 4.4 Conclusion

In conclusion, our survey results suggest that multiple factors influence doorway freezing. As expected, narrow doorways are more likely to increase freezing. However, other factors are also at play, such as clutter around doorways, lighting conditions and automatic doors. We provided recommendations on how to adapt doorways in daily life settings. Of note, the same but opposite recommendations could be used to increase freezing likelihood in laboratory experiments or in clinical evaluations. Remarkably, some doorway characteristics induced opposite responses in different people and speaking to people about their personal experiences with doorway freezing can add valuable information. Finally, we suggest that doorway freezing has underlying causes also in the attentional and emotional domains, rather than being purely a visuomotor issue.

## Data availability statement

The original contributions presented in the study are publicly available. This data can be found on the Donders Repository: https://doi.org/10.34973/dfcs-4194. The analysis code related to this article is available on Github: https://github.com/helenacockx/doorway_characteristics.

## Ethics statement

The studies involving humans were approved by Research Ethics Committee of the Faculty of Science of the Radboud University. The studies were conducted in accordance with the local legislation and institutional requirements. The participants provided their written informed consent to participate in this study.

## Author contributions

HC: Conceptualization, Data curation, Methodology, Supervision, Writing – original draft. EL: Conceptualization, Data curation, Formal analysis, Methodology, Visualization, Writing – original draft. RW: Conceptualization, Funding acquisition, Supervision, Writing – review & editing. IC: Conceptualization, Funding acquisition, Supervision, Writing – review & editing.

## Footnotes

^1^ https://www.parkinsonnext.nl/overons/
