# Supplementary material for: The effect of doorway characteristics on freezing of gait in Parkinson’s disease
Source: Front Neurol. 2023 Dec 4;14:1265409. doi: 10.3389/fneur.2023.1265409 (PMC10726031; doi:10.3389/fneur.2023.1265409)
Supplement: Supplementary file 1 [file Table_1.DOCX]

**Supplementary Table 1. Answers to the open question “Are there any other doorway characteristics that influence your freezing?” per doorway characteristic**

| **Doorway characteristics** | **Number of mentions** |
| --- | --- |
| Doorhandles in the same spot  Light shining on the floor  Slightly open sliding door  People or objects standing near the door  Light temperature  Person walking behind you  Resistance when closing or opening  When the door is in a corner  Sliding door without guiding rails  When there are no people around  Automatic doors that close quickly  Tile pattern in the next room | 1  1  1  4  1  1  4  1  1  1  1  2 |
