# Supplementary material for: The effect of doorway characteristics on freezing of gait in Parkinson’s disease
Source: Front Neurol. 2023 Dec 4;14:1265409. doi: 10.3389/fneur.2023.1265409 (PMC10726031; doi:10.3389/fneur.2023.1265409)
Supplement: Supplementary file 2 [file Data_Sheet_1.DOCX]

**Inleiding vragenlijst**

Hartelijk bedankt voor uw interesse in deze vragenlijst. De vragenlijst richt zich op “Freezing of Gait” (bevriezen van het lopen) bij mensen met de ziekte van Parkinson. Met *freezing* bedoelen we het gevoel alsof u (kortdurend) met de voeten aan de grond genageld, of ‘vastgeplakt’, staat. Deze vragenlijst helpt ons inschatten in welke situaties mensen last hebben van freezing.

Afhankelijk van uw antwoorden in het eerste deel van de vragenlijst, kan het zijn dat we u vragen of we u een vervolgvragenlijst mogen sturen. Specifiek heeft die vervolgvragenlijst het doel een duidelijker beeld te schetsen over het optreden van freezing bij deuropeningen. Hierbij onderzoeken we welke kenmerken van een deuropening invloed hebben op freezing bij het passeren van de deur. Dit kan ons helpen verzorginstehuizen, openbare plaatsen of uw eigen woning beter in te richten met deuren die zo min mogelijk freezing uitlokken. Daarmee kan ook het risico op valincidenten worden verminderd. Daarnaast kan de vragenlijst ons helpen meer inzicht te krijgen in de onderliggende mechanismen van freezing bij deuren.

Samengevat vragen we u dus om één of twee vragenlijsten in te vullen. De eerste vragenlijst gaat over freezing in het algemeen, en vragen we u ook in te vullen als u geen last heeft van freezing bij deuren. Deze eerste vragenlijst zal ongeveer 10 minuten in beslag nemen. Als u last heeft van freezing bij deuren krijgt u vervolgens een uitnodiging om daar een vervolgvragenlijst over in te vullen. Deze vervolgvragenlijst sturen we u één tot twee weken na het voltooien van deze eerste vragenlijst toe.

De gegevens die worden verzameld tijdens het invullen van de vragenlijsten, zullen gecodeerd worden opgeslagen. Dit houdt in dat alle gegevens die herleidbaar zijn naar u als persoon zullen worden vervangen door een niet-herleidbare code. Herleidbare gegevens zullen niet beschikbaar zijn voor de onderzoekers.

**Toestemmingsverklaring**

Om mee te doen aan dit vragenlijstonderzoek dient u akkoord te gaan met onderstaande toestemmingsverklaring.

Ik verklaar hierbij dat:

- Ik duidelijk ben geïnformeerd over het doel en de methode van dit onderzoek waaraan ik vrijwillig zal deelnemen.
- Ik weet dat mijn gegevens gecodeerd worden en niet herleidbaar zijn tot mij als persoon. Ik geef toestemming voor het verzamelen, gebruiken en bewaren van deze gecodeerde gegevens.
- Ik ben me ervan bewust dat ik het recht heb om deze toestemming in te trekken en dat ik de vragenlijst voortijdig kan beëindigen zonder daar een reden voor op te hoeven geven. De gegevens die tot dat moment verzameld zijn, kunnen wel gebruikt worden voor het onderzoek

Bij vragen mag ik contact opnemen Eefke Lemmen (email xxx, telefoonnummer: xxx).

*Indien niet akkoord:*

Hier eindigt voor u de vragenlijst. Bedankt voor uw interesse en tijd.

*Indien akkoord:*

Om het maximale te bereiken met de gegevens die wetenschappers verzamelen, worden er steeds meer van deze gegevens openbaar gedeeld. Op die manier kunnen onderzoekers binnen hetzelfde vakgebied elkaars gegevens gebruiken, waardoor er zo veel mogelijk informatie uit gehaald kan worden, en er geen dubbel werk gedaan hoeft te worden. Graag willen wij de resultaten van deze vragenlijst daarom kunnen delen met anderen. Uiteraard worden alleen de gecodeerde gegevens gedeeld, die op geen enkele manier te herleiden zijn tot u als persoon. Geeft u toestemming voor het delen van uw gegevens?

- Ja
- Nee

Hierna volgen een aantal vragen voorafgegaan door een korte instructie.

De vragenlijst zal ongeveer 10 minuten in beslag nemen.

**Ter instructie**
Er bestaat soms onduidelijkheid over wat freezing precies is. Met freezing bedoelen we het gevoel dat u met de voeten aan de grond genageld of geplakt staat. Freezing kan gepaard gaan met trillende benen en het maken van kleine schuifelpasjes. U kunt er bijvoorbeeld last van krijgen wanneer u aanstalten maakt om te gaan lopen, wanneer u een bocht maakt of wanneer u door een smalle doorgang of in een ruimte met veel andere mensen loopt. Weet u niet zeker of u last heeft van freezing? Kijk dan eens naar [deze](https://www.youtube.com/watch?v=EP30xJDk2S8) video over freezing. Eventuele vragen, op- of aanmerkingen kunt u bij de vraag of helemaal aan het eind van de vragenlijst achterlaten, mailen naar [xxx](mailto:eefke.lemmen@ru.nl), of bellen naar xxx.

1. **Heeft u de ‘gewone’ ziekte van Parkinson (ook wel ‘idiopathische’ ziekte van Parkinson genoemd) of een andere vorm van parkinsonisme?**

- Ik heb de ‘gewone’ ziekte van Parkinson
- Ik heb een andere vorm van parkinsonisme, namelijk:
  - Progressieve supranucleaire verlamming (PSP)
  - Multipele systeem atrofie (MSA)
  - Corticobasale degeneratie (CBDG)
  - Vasculair Parkinsonisme
  - Medicamenteus Parkinsonisme
  - Lewy body dementie (LBD)
  - Anders: .

1. **Hoeveel jaar geleden heeft u de diagnose van de ziekte van Parkinson gekregen?**

|  |
| --- |

1. **Wat is uw geslacht?**

- Man
- Vrouw
- Anders

1. **Wat is uw leeftijd?**

|  |
| --- |

1. **Hebt u in de afgelopen maand last gehad van freezing?**

- Nee *🡪 Einde vragenlijst*
- Ja

1. **Hoe vaak ervaart u freezingepisoden?**

- Minder dan één keer per week
- Weinig, ongeveer één keer per week
- Dikwijls, ongeveer één keer per dag
- Heel dikwijls, meer dan één keer per dag

1. **Hoe vaak ervaart u freezingepisoden terwijl u draait?**

- Nooit *🡪 Ga verder naar vraag 9*
- Heel zelden, ongeveer één keer per maand
- Weinig, ongeveer één keer per week
- Dikwijls, ongeveer één keer per dag
- Heel dikwijls, meer dan één keer per dag

1. **Hoe lang duurt uw langste freezingepisode bij het draaien?**

- Heel kort: 1 seconde
- Kort, 2-5 seconden
- Lang, tussen de 5 en 30 sec
- Zeer lang, niet in staat om binnen 30 seconden weer te gaan (lopen)

1. **Hoe vaak ervaart u freezingepisoden als u de eerste stap zet om te gaan (lopen)?**

- Nooit *🡪 Ga verder naar vraag 11*
- Heel zelden, ongeveer één keer per maand
- Weinig, ongeveer één keer per week
- Dikwijls, ongeveer één keer per dag
- Heel dikwijls, meer dan één keer per dag

1. **Hoe lang duurt uw langste freezingepisode als u de eerste stap zet om te gaan (lopen)?**

- Heel kort, 1 sec
- Kort, 2-5 sec
- Lang, tussen de 5 en 30 seconden
- Zeer lang, niet in staat om binnen 30 seconden te stappen

1. **Hoe vaak ervaart u freezingepisoden als u door een deuropening loopt?**

- Nooit *🡪 Ga verder naar vraag 13*
- Heel zelden, ongeveer één keer per maand
- Weinig, ongeveer één keer per week
- Dikwijls, ongeveer één keer per dag
- Heel dikwijls, meer dan één keer per dag

1. **Hoe lang duurt uw langste freezingepisode als u door een deuropening loopt?**

- Heel kort, 1 sec
- Kort, 2-5 sec
- Lang, tussen de 5 en 30 seconden
- Zeer lang, niet in staat om binnen 30 seconden te stappen

1. **Hoe storend zijn de freezingepisoden voor uw dagelijks leven?**

- Helemaal niet
- Weinig
- Matig
- Erg

1. **Veroorzaken de freezingepisoden gevoelens van onzekerheid of angst om te vallen?**

- Helemaal niet
- Weinig
- Matig
- Erg

1. **Beperken de freezingepisoden uw dagelijkse activiteiten?**

*(scoor alleen de impact van freezing op uw dagelijkse activiteiten. Niet de impact van de ziekte in het algemeen)*

- Helemaal niet, ik voer taken zoals normaal uit
- Weinig, ik vermijd slechts sommige taken
- Matig, ik vermijd een substantieel deel (ongeveer de helft) van mijn dagelijkse activiteiten
- Belangrijk, ik ben heel beperkt in het uitvoeren van de meeste dagelijkse activiteiten

1. **Geef aan hoe u zich voelt tijdens het lopen door een deur:**

| **Tijdens het lopen door een deur** | Helemaal niet | Niet zo veel | Redelijk Veel | Veel | Erg veel |
| --- | --- | --- | --- | --- | --- |
| Voel ik dat ik me moet inspannen |  |  |  |  |  |
| Maak ik me zorgen over wat andere mensen denken over mijn bewegingen |  |  |  |  |  |
| Denk ik aan andere momenten waarop ik mijn balans verloor |  |  |  |  |  |
| Denk ik aan wat er zou gebeuren als ik zou vallen |  |  |  |  |  |
| Raak ik in de war en maak ik onlogische keuzes |  |  |  |  |  |
| Gaan er zorgelijke gedachten over vallen door mijn hoofd |  |  |  |  |  |
| Probeer ik na te denken over de manier waarop ik loop/beweeg |  |  |  |  |  |
| Probeer ik bewust mijn bewegingen te controleren |  |  |  |  |  |
| Observeer ik de manier waarop ik loop/beweeg |  |  |  |  |  |
| Voel ik me gespannen |  |  |  |  |  |
| Vind ik het moeilijk om me op twee dingen tegelijk te concentreren |  |  |  |  |  |

U bent aan het einde gekomen van deze vragenlijst. Wanneer er nog eventuele op- of aanmerkingen zijn over deze vragenlijst, kunt u dat hieronder invullen:

**Voorbereiding vragenlijst II**

In deze vragenlijst heeft u aangegeven minimaal 1 keer per week last te hebben van freezing bij een deuropening. Daarom willen we u graag vragen een tweede vragenlijst over freezing bij deuren in te vullen. Deze vragenlijst gaat over kenmerken van een deur die freezing kunnen verergeren, of juist verminderen.

Om u hierop voor te bereiden vragen we u een week lang foto’s te maken van deuren waarbij u vaker dan normaal last heeft van freezing, en deuren waarbij u minder vaak last heeft van freezing. Daarnaast nodigen we u uit met mensen uit uw omgeving te praten over freezing bij deuren. Vaak zien zij dingen die u zelf nog niet zijn opgevallen. Het maken van de foto’s en praten over freezing bij deuren zal ervoor zorgen dat het invullen van de vervolgvragenlijst makkelijker voor u is, aangezien u op deze manier al na heeft gedacht over welke kenmerken van deuren freezing verergeren of verminderen. Als u deelneemt aan het tweede deel van dit onderzoek, zullen we u bovenstaande instructie ook per email sturen.

Als u besluit deel te nemen aan het tweede deel van dit onderzoek, vragen wij u een emailadres op te geven. Dit emailadres gebruiken wij alleen om u de volgende vragenlijst toe te sturen en zal verder niet bewaard worden. Hierop ontvangt u dan op datum x een uitnodiging om de vragenlijst in te vullen. Mocht u op, of kort na deze datum geen tijd hebben om de vragenlijst in te vullen, dat krijgt u een week later nog een herinneringsmail. De onderzoekers kunnen uw antwoorden niet koppelen aan uw emailadres, waardoor uw privacy beschermd wordt. Deze vragenlijst zal ongeveer 20 minuten in beslag nemen.

1. **Heeft u interesse om deel te nemen aan het vervolgonderzoek?**

- Ja *🡪 Ga verder naar vraag 2*
- Nee *🡪 Ga verder naar vraag 3*

1. **Op welk emailadres mogen we u op de aangegeven datum de vragenlijst toesturen?**
2. **Waarom wilt u niet deelnemen aan het vervolgonderzoek?**
   - Dat zeg ik liever niet
   - Ik vind het niet prettig om mijn contactinformatie op te geven
   - Het invullen duurt te lang
   - De vragen zijn te moeilijk
   - De voorbereiding neemt te veel tijd in beslag
   - De voorbereiding is te moeilijk

**Vragenlijst II**

Op datum x heeft u het eerste deel van onze vragenlijst over freezing ingevuld. Uit uw antwoorden is gebleken dat u wel eens last heeft van freezing bij het passeren van deuren. Daarom bent u uitgenodigd om deze vervolgvragenlijst in te vullen. Hiermee kunnen we inzicht krijgen welke eigenschappen van een deur de kans op freezing kunnen vergroten, of juist verkleinen.

Uit eerder onderzoek blijken de uitlokkers voor freezing rond deuropeningen te kunnen verschillen tussen personen. Waar de één bijvoorbeeld meer last van freezing heeft bij het lopen door deuropeningen met een drempel, heeft de ander meer last bij hele smalle deuren.

Afgelopen week heeft u foto’s gemaakt van deuropeningen waarbij u veel, of juist weinig last heeft van freezing. Deze foto’s kunt u gebruiken ter ondersteuning bij het invullen van de vragenlijst. Daarnaast heeft u met mensen uit uw omgeving gesproken over hun indruk van uw freezing bij deuren. Die ervaringen mag u meenemen bij het invullen van de vragenlijst. Ook als u de afgelopen week geen foto’s heeft gemaakt kunt u de vragenlijst invullen. Uiteraard mag u ook tijdens het invullen nog met iemand overleggen.

Deze vragenlijst zal ongeveer 20 minuten in beslag nemen. Aan het einde van de vragenlijst is ruimte om kenmerken van deuropeningen aan te geven die niet in onze vragenlijst voorkomen, maar wel invloed hebben op uw freezing, en voor eventuele andere opmerkingen, vragen, of aanvullingen. Bij vragen mag u contact opnemen Eefke Lemmen (email: [xxx](mailto:eefke.lemmen@student.ru.nl), telefoonnummer: xxx).

Om deze vragenlijst te koppelen aan de eerder ingevulde vragenlijst, vragen we u hieronder uw e-mailadres in te vullen. Dit e-mailadres wordt door de medewerkers van ParkinsonNEXT gebruikt om de gegevens van beide vragenlijsten aan elkaar te kunnen koppelen. Voordat de gegevens naar de onderzoekers van dit project worden gedeeld zal het e-mailadres geanonimiseerd worden. Deze vragenlijst is daarna niet meer herleidbaar naar u als persoon.

Hieronder noemen we een aantal kenmerken van deuren, die invloed kunnen hebben op freezing, zoals hoogte van de deur, of de aanwezigheid van een drempel. Voor elk kenmerk vult u op een schaal van -2 tot +2 in of u minder of meer freezing ervaart in vergelijking met een gemiddelde deur.Tenzij anders vermeld mag u er vanuit gaan dat de deur al open staat. Als uw freezing niet meer, maar ook niet minder wordt bij een bepaald kenmerk, kiest u voor het midden van de schaal (0).

Als u bijvoorbeeld veel minder freezing ervaart bij een smalle deur, vergeleken met een gemiddelde deur, dan vult u bij die vraag -2 in. Als u bij een smalle deur net zo veel freezing ervaart als bij een gemiddelde deur, dan vult u 0 in. Als u bij een smalle deur veel meer freezing heeft dan bij een gemiddelde deur, dan vult u +2 in. Als u niet weet wat voor invloed een kenmerk heeft op uw freezing, mag u die vraag overslaan. U hoeft dus nergens verplicht een antwoord in te vullen.

1. **Ervaart u, vergeleken met een gemiddelde, openstaande deur meer of minder freezing bij**

Blok 1

**Een smalle deur**

Veel minder freezing -2 -1 0 1 2 Veel meer freezing

**Een brede deur**

Veel minder freezing -2 -1 0 1 2 Veel meer freezing

**Een lage deur**

Veel minder freezing -2 -1 0 1 2 Veel meer freezing

**Een hoge deur**

Veel minder freezing -2 -1 0 1 2 Veel meer freezing

**Een gesloten deur**

Veel minder freezing -2 -1 0 1 2 Veel meer freezing

**Een gesloten deur die u *naar u toe moet* openen**

Veel minder freezing -2 -1 0 1 2 Veel meer freezing

**Een gesloten deur die u *van u af* moet openen**

Veel minder freezing -2 -1 0 1 2 Veel meer freezing

**Een doorzichtige deur**

Veel minder freezing -2 -1 0 1 2 Veel meer freezing

**Als u vlak *voor* de deur een bocht moet maken**

Veel minder freezing -2 -1 0 1 2 Veel meer freezing

**Als u meteen *na* de deur een bocht moet maken**

Veel minder freezing -2 -1 0 1 2 Veel meer freezing

Blok 2

**Ervaart u, vergeleken met een gemiddelde, openstaande deur, meer of minder freezing bij**

**Een deur met een drempel**

Veel minder freezing -2 -1 0 1 2 Veel meer freezing

**Wanneer de vloerbedekking voor en na de deur er anders uitziet**

Veel minder freezing -2 -1 0 1 2 Veel meer freezing

**Wanneer er een deurmat bij de deur ligt**

Veel minder freezing -2 -1 0 1 2 Veel meer freezing

**Wanneer er een opstapje bij de deur is**

Veel minder freezing -2 -1 0 1 2 Veel meer freezing

**Wanneer er een afstapje bij de deur is**

Veel minder freezing -2 -1 0 1 2 Veel meer freezing

Blok 3

**Ervaart u, vergeleken met een gemiddelde, openstaande deur, meer of minder freezing bij**

**Als u onbekend bent met de deur**

Veel minder freezing -2 -1 0 1 2 Veel meer freezing

**Als u onbekend bent met de ruimte achter de deur**

Veel minder freezing -2 -1 0 1 2 Veel meer freezing

**Wanneer de ruimte na de deur klein is**

Veel minder freezing -2 -1 0 1 2 Veel meer freezing

**Wanneer de ruimte na de deur groot is**

Veel minder freezing -2 -1 0 1 2 Veel meer freezing

**Wanneer de deur zich in een smalle gang/ruimte bevindt**

Veel minder freezing -2 -1 0 1 2 Veel meer freezing

**Wanneer de deur zich in een brede gang/ruimte bevindt**

Veel minder freezing -2 -1 0 1 2 Veel meer freezing

**Wanneer de ruimte *na* de deur erg druk of vol is**

Veel minder freezing -2 -1 0 1 2 Veel meer freezing

**Wanneer de ruimte *voor* de deur erg druk of vol is**

Veel minder freezing -2 -1 0 1 2 Veel meer freezing

**Wanneer de muur waarin de deur zich bevindt druk is**

Veel minder freezing -2 -1 0 1 2 Veel meer freezing

**Als de ruimte na de deur donkerder is dan de ruimte voor de deur**

Veel minder freezing -2 -1 0 1 2 Veel meer freezing

**Als de ruimte na de deur lichter is dan de ruimte voor de deur**

Veel minder freezing -2 -1 0 1 2 Veel meer freezing

Blok 4

**Ervaart u, vergeleken met een gemiddelde, openstaande deur, meer of minder freezing bij**

**Een automatische schuifdeur**

Veel minder freezing -2 -1 0 1 2 Veel meer freezing

**Wanneer u een automatische draaideur *in* moet stappen**

Veel minder freezing -2 -1 0 1 2 Veel meer freezing

**Wanneer u een automatische draaideur *uit* moet stappen**

Veel minder freezing -2 -1 0 1 2 Veel meer freezing

**Wanneer u meerdere deuren na elkaar moet passeren**

Veel minder freezing -2 -1 0 1 2 Veel meer freezing

**Zijn er nog andere eigenschappen van een deur die invloed hebben op uw freezing? Vul deze dan hieronder in, en vermeld daarbij of ze zorgen voor minder of meer freezing. U kunt hiervoor de foto’s gebruiken die u voorbije week genomen hebt.**

**U bent aan het einde gekomen van deze vragenlijst. Wanneer er nog eventuele op- en aanmerkingen zijn over deze vragenlijst, kunt u dat hieronder invullen:**
